# Patient characteristics from a Melbourne based adult eating disorders inpatient unit

**DOI:** 10.1186/2050-2974-1-S1-P7

**Published:** 2013-11-14

**Authors:** Sarah Mitchell, Lisa Stokes

**Affiliations:** 1 Eating Disorders Unit, NorthWestern Mental Health, Royal Melbourne Hospital, Australia

## 

This study aims to inform clinicians and researchers about the characteristics of patients admitted to the Eating Disorders Inpatient Unit, NorthWestern Mental Health, Royal Melbourne Hospital across a 4 year period. It is anticipated that this information can provide insights into the potential base rates for particular inpatient diagnosis, chronicity of illness, duration of admissions and planned versus unplanned discharges. Data was collected retrospectively via a hospital file audit of admissions to the unit between the years 2009 and 2012. Across the 4 year period there were a total of 193 admissions to the unit and just over a third of patients (35%) were placed on an involuntary treatment order. On admission, the average eating disorder duration was 5.4 years and eating disorder diagnosis comprised; anorexia nervosa-restricting type (65.8%), anoirexia nervosa–binge purge type (6.8%), bulimia Nervosa (3.2%), eating disoirdfer noit otherwise specified (18.9%) and no eating disorder diagnosis (4.7%). The average duration of admission was 36 days and the majority of discharges were planned (88.4%) compared to unplanned (11.6%). Given the chronicity of illness, these findings emphasise the importance of intensive follow-up given the relatively short inpatient stay which may include outreach services, frequent individual/group outpatient appointments and utilising day patient programs as a step down following discharge.

